# Supplementary material for: Infant and young child feeding practices among mothers in the pilot Micronutrient Powder Initiative in four geographically and ethnically diverse districts in Ghana
Source: PLoS One. 2024 Aug 1;19(8):e0307961. doi: 10.1371/journal.pone.0307961 (PMC11293642; doi:10.1371/journal.pone.0307961)
Supplement: S1 File — (DOC) [file pone.0307961.s001.doc]

**Lessons learned from the use of Micronutrient Powders in young child feeding in four districts in Ghana**

**Appendix 1: Questionnaire for Staff of Ghana Health Service**

**Respondent**: Please provide a numerical rating (**1 = Not at All or Poor; 2 = Adequate or Satisfactory; and 3 = To a great extent or Excellent**) for each of the questions. As much as possible, please always provide comments on the

| Staff of (Organization): _______________________ | | | | | | Date (dd/mm/yyyy): ________________ | |  |
| --- | --- | --- | --- | --- | --- | --- | --- | --- |
|  | |  | |  | |  | |  |
|  | | **QUESTIONS** | | **RATING** | | **COMMENTS/RESPONSE** | |  |
|  | | **SUMMARY** | | **OF ACTIVITIES/DECISIONS** | | | |  |
| 1) | | What sequence of activities did you perform | |  | |  | |  |
|  | | during the implementation of the project? | |  | |  | |  |
| 2) | | What key decisions did you take during the | |  | |  | |  |
|  | | implementation of the project? | |  | |  | |  |
|  | | **PROJECT EFFECTIVENESS** | |  | |  | |  |
| 3) | | To what extent were the objectives of the project | |  | |  | |  |
|  | | met? | |  | |  | |  |
|  | |  | |  | |  | |  |
| 4) | | What is your overall assessment of the outcome of | |  | |  | |  |
|  | | this project? | |  | |  | |  |
|  | | **SCOPE AND** | | | | **SCHEDULE** | |  |
| 5) | | How satisfied are you with your involvement in the | |  | |  | |  |
|  | | development of the project scope during project | |  | |  | |  |
|  | | initiation and planning? | |  | |  | |  |
| 6) | | How closely did the initial project schedule | |  | |  | |  |
|  | | compare with the actual schedule? | |  | |  | |  |
| 7) | | How did the estimated project budget compare | |  | |  | |  |
|  | | with the total actual expenditures? | |  | |  | |  |
| 8) | | How effective were Best Practices & Lessons | |  | |  | |  |
|  | | Learned from prior projects used in this project? | |  | |  | |  |
|  | | **COMMUNICATION MANAGEMENT** | | | | | |  |
| 9) | | How effective were the communication materials | |  | |  | |  |
|  | | in providing and orienting district/hospital staff | |  | |  | |  |
|  | | involved in the project about the details of the | |  | |  | |  |
|  | | project? | |  | |  | |  |
| 10) | | How efficient were project team meetings | |  | |  | |  |
|  | | conducted? | |  | |  | |  |
| 11) | | How timely were progress reports provided to the | |  | |  | |  |
|  | | Project Manager by Team Members? | |  | |  | |  |
| 12) | | How actively and meaningfully were stakeholders | |  | |  | |  |
|  | | involved in the project? | |  | |  | |  |
| 13) | | Were stakeholder communications adequate and | |  | |  | |  |
|  | | effective? | |  | |  | |  |
| 14) | | How well was project status communicated | |  | |  | |  |
|  | | throughout your involvement in the project? | |  | |  | |  |
| 15) | | How well were project issues communicated | |  | |  | |  |
|  | | throughout your involvement in the project? | |  | |  | |  |
| 16) | | How well did the Project Manager respond to your | |  | |  | |  |
|  | | questions or comments related to the project? | |  | |  | |  |
|  | |  | | **ISSUE MANAGEMENT** | | | |  |
| 17) | | How effectively were issues managed on the | |  | |  | |  |
|  | | project? | |  | |  | |  |
| 18) | | How effectively were issues resolved before | |  | |  | |  |
|  | | escalation was necessary? | |  | |  | |  |
| 19) | | How effectively were issues able to be resolved | |  | |  | |  |
|  | | without impacting the Project Schedule? | |  | |  | |  |
|  | | **PROJECT** | | **IMPLEMENTATION & SUPPORT** | | | |  |
| 20) | | How effective was the documentation that you | |  | |  | |  |
|  | | received with the project implementation? | |  | |  | |  |
| 21) | | How effective was the training you received in | |  | |  | |  |
|  | | preparation for the project? | |  | |  | |  |
| 22) | | How timely was the training you received in | |  | |  | |  |
|  | | preparation for the project? | |  | |  | |  |
| 23) | | How effective was the support you received during | |  | |  | |  |
|  | | implementation of the project? | |  | |  | |  |
|  | | **PERFORMANCE OF THE PROJECT TEAM** | | | | | |  |
| 24) | | How well did the Project Team understand the | |  | |  | |  |
|  | | expectations of their specific roles and | |  | |  | |  |
|  | | responsibilities? | |  | |  | |  |
| 25) | How well were your expectations met regarding | |  | |  | |  | |
|  | the extent of your involvement in the project | |  | |  | |  | |
|  | (effort, time commitments, etc.)? | |  | |  | |  | |
| 26) | How effective was each Project Team member in | |  | |  | |  | |
|  | fulfilling his/her role? | |  | |  | |  | |
|  |  | | **GENERAL QUESTIONS** | | | |  | |
| 27) | What were the most important issues on this | |  | |  | |  | |
|  | project? | |  | |  | |  | |
| 28) | What went well as planned or intended? | |  | |  | |  | |
| 29) | What did not go well as planned or intended? | |  | |  | |  | |
| 30) | What unintended consequences were observed? | |  | |  | |  | |
| 31) | What important problems did you have to dealt | |  | |  | |  | |
|  | with at any time during the project; what innovative | |  | |  | |  | |
|  | strategies did you put in place to address those | |  | |  | |  | |
|  | problems. | |  | |  | |  | |
| 32) | What would you do differently, if the project was to | |  | |  | |  | |
|  | be implemented all over again? | |  | |  | |  | |
| 33) | What were the lessons learned on this project? | |  | |  | |  | |
| 34) | What recommendations or advice would you offer | |  | |  | |  | |
|  | to others implementing a similar program on a | |  | |  | |  | |
|  | pilot basis and on a larger scale? | |  | |  | |  | |

**UP Template Version:** 11/30/06 Page 2 of 2

Source: CDC (https://www2.cdc.gov/cdcup/library/templates/CDC_UP_Lessons_Learned_Post_[Project_Survey.doc)](https://www2.cdc.gov/cdcup/library/templates/CDC_UP_Lessons_Learned_Post_Project_Survey.doc)

**Lessons learned from the use of Micronutrient Powders in young child feeding in four districts in Ghana Appendix 2: Key Informants Interview Guide**

**-------------------------------------------------------------------------------------------------------------------------------------------------------------------------------------------------------------------------------**

Opening Section

| 1. Please tell me about your influence in your | | Response: |
| --- | --- | --- |
| community. | |  |
| a. | Probe for influence related to nutrition and IYCF |  |
|  | (EBF/CF/ Continued BF |  |
| b. | What do you think community members see as your |  |
|  | influence? |  |
| c. | Connections with NGOs, GHS, CHIP Centers, |  |
|  | community groups |  |
|  |  |  |

Use of Infant and Young Child Feeding (IYCF) guidelines in community I would like to ask you about IYCF practices in your community.

| 2. To what extent do women in your community practice | |  |  |
| --- | --- | --- | --- |
| the recommended IYCF guidelines. Provide a | | IYCF guideline | Rating Comment |
| numerical rating (**1=Not at All or Poor; 2=Adequate or** | | Early Initiation of B/F (within 1 hr) |  |
| **Satisfactory; and 3=To a great extent or Excellent**) and | | EBF for first 6 mo |  |
| a comment for each of the IYCF guidelines. | | Timely intro of CFd (after 6 mo) |  |
|  |  | Cont'd BF for 2 yr or beyond |  |
|  |  | Age approp CF ( |  |
|  |  | 4 or more food groups |  |
|  |  | 6‐8 mo (breastfed) – 2x |  |
|  |  | 9‐23 mo (breastfed) – 3x |  |
|  |  | 6‐23 mo (non‐breastfed) – 4x |  |
|  |  | Active feeding during/after illness |  |
| 3. What are common beliefs around breastfeeding | | Response: |  |
| and infant care practices in this community? | |  |  |
| a. | Beliefs- Colostrum: Is colostrum fed to babies? What |  |  |
|  | do people think about colostrum? |  |  |
| b. | Beliefs- first feeding: Should baby be given anything |  |  |
|  | (honey, water, medicine, tea etc) before being |  |  |
|  | breastfed for the first time? |  |  |
| c. | Beliefs-Feeding in first days of life: What should |  |  |
|  | infants eat in first few days of life? |  |  |
| d. | Insufficient milk- How common is insufficient milk? |  |  |
| e. | Beliefs-insufficient milk: Why do mothers think they |  |  |
|  | have insufficient milk? What may be causing mothers |  |  |
|  | to think that they have insufficient milk? |  |  |
| f. | Beliefs-exclusive breastfeeding: Do they think mother |  |  |
|  | would be able to only give breastmilk for the first 6 |  |  |
|  | months? What would mothers need to be able to do |  |  |
|  | this? |  |  |
| g. | Time/Availability-Are mothers available to |  |  |
|  | breastfeed their child? Why? Why not? |  |  |
| h. | Perceived positive consequences- Do people think |  |  |
|  | that giving other foods (water, tea, etc) may be |  |  |
|  | beneficial to child health |  |  |
| i. | Perceived negative consequences-May not be viewed |  |  |
|  | as serious not to feed child other foods |  |  |
|  | |  |  |
| 4. In your opinion, which breastfeeding | | a. Why |  |
| recommendations are hardest for women to | |  |  |
| follow? | |  |  |
|  |  |  |  |

1

| 5. In your opinion what influences the way | | Response: |
| --- | --- | --- |
| women practice IYCF guidelines in the | |  |
| community? | |  |
| a. | Knowledge-what do they feed to children? How |  |
|  | much? When? Diversity? |  |
| b. | Beliefs- water: When should water be |  |
|  | introduced after birth? |  |
| c. | Beliefs-animal milks: When should animal milks |  |
|  | first be introduced? |  |
| d. | Beliefs-porridge: When should porridge first be |  |
|  | introduced? What should the consistency of |  |
|  | porridge be? How is it fed (spoon, bottle, cup) |  |
| 6. What support (community, institutional, etc) | | Response: |
| do women receiving concerning IYCF? | |  |
| 7. In your opinion, which complementary feeding | | d. Why? |
| recommendation is hardest for women to | |  |
| follow? | |  |
| •Early Initiation of B/F (within 1 hr) | |  |
| •EBF for first 6 mo | |  |
| •Timely intro of CFd (after 6 mo) | |  |
| •Cont'd BF for 2 yr or beyond | |  |
| •Age approp CF ( | |  |
| Questions related to the MNP distribution and use | | |
|  |  |  |
| 8. What do you know about the MNPs given to | | Response: |
|  |
| mothers to add to their children’s food? | |  |
| •How was the MNP Initiative introduced (How did | |  |
|  | women get to know, and start receiving the MNPs)? |  |
| •What did the mothers in the community think about | |  |
|  | the MNP program? |  |
| •What did the health facilities staff did whilst running | |  |
|  | the program? |  |
| •What actions or approaches taken by the health | |  |
|  | workers went well? |  |
| •Which actions or approaches taken by the health | |  |
|  | workers did not go well? |  |
| •What examples of unexpected results did women | |  |
|  | encounter? |  |
| •What are women’s future expectations? | |  |

2

**Lessons learned from the use of Micronutrient Powders in young child feeding in four districts in Ghana**

**Appendix 3: Focus Group Discussion (FGD) Guide**

**-------------------------------------------------------------------------------------------------------------------------------------------------------------------------------------------------------------------------------**

Opening Section

First, please let’s all introduce ourselves by telling a bit about ourselves, for example about your children and family. [*Go around and* *introduce – Notetaker should take detailed notes on how they describe themselves, without capturing names*]

Use of Infant and Young Child Feeding (IYCF) guidelines in community I would like to ask you about IYCF practices in your community.

1. To what extent do women in your community practice

| the recommended IYCF guidelines. Provide a | IYCF guideline | Rating Comment |
| --- | --- | --- |
| numerical rating (**1=Not at All or Poor; 2=Adequate or** | Early Initiation of B/F (within 1 hr) |  |
| **Satisfactory; and 3=To a great extent or Excellent**) and | EBF for first 6 mo |  |
| a comment for each of the IYCF guidelines. | Timely intro of CFd (after 6 mo) |  |
|  | Cont'd BF for 2 yr or beyond |  |
|  | Age approp CF ( |  |
|  | 4 or more food groups |  |
|  | 6‐8 mo (breastfed) – 2x |  |
|  | 9‐23 mo (breastfed) – 3x |  |
|  | 6‐23 mo (non‐breastfed) – 4x |  |
|  | Active feeding during/after illness |  |

1. Breastfeeding and introduction to complementary feeding
   - In this community, how are babies fed on first day of life?
   - What are the first liquids/foods other than breastmilk that are given?
   - What are some reasons that mothers might introduce liquids and/or foods other than breastmilk before 6 months?
   - What are some reasons that mothers might wait until after 6 months to introduce liquids and/or foods other than breastmilk?
   - What are some common foods given to infants around 6-8 months?
   - Are there any foods that are not appropriate for infants 6-8 months? If so, what are they and why are they not appropriate?
   - What are common foods or drinks that infants take from 9-24 months?
   - Are there any foods that are not appropriate for children at this age? What are they and why are they not appropriate?
   - Are there any differences in how sons and daughters are fed in this community? If so, what are they and why?

3. What are common beliefs around breastfeeding Response:

and infant care practices in this community?

1. Beliefs- Colostrum: Is colostrum fed to babies? What do people think about colostrum?
2. Beliefs- first feeding: Should baby be given anything (honey, water, medicine, tea etc) before being breastfed for the first time?
3. Beliefs-Feeding in first days of life: What should infants eat in first few days of life?
4. Insufficient milk- How common is insufficient milk?
5. Beliefs-insufficient milk: Why do mothers think they have insufficient milk? What may be causing mothers to think that they have insufficient milk?
6. Beliefs-exclusive breastfeeding: Do they think mother would be able to only give breastmilk for the first 6 months? What would mothers need to be able to do this?
7. Time/Availability-Are mothers available to breastfeed their child? Why? Why not?
8. Perceived positive consequences- Do people think that giving other foods (water, tea, etc) may be

|  | beneficial to child health | 1 |
| --- | --- | --- |
| i. | Perceived negative consequences-May not be viewed |  |
|  | as serious not to feed child other foods |  |

**Lessons learned from the use of Micronutrient Powders in young child feeding in four districts in Ghana**

|  | **Appendix 3: Focus Group Discussion (FGD) Guide** | |
| --- | --- | --- |
|  | **-------------------------------------------------------------------------------------------------------------------------------------------------------------------------------------------------------------------------------** | |
| 4. | In your opinion, which breastfeeding | a. Why |
|  | recommendations are hardest for women to |  |
|  | follow? |  |
|  |  |  |
| 5. | What support (community, institutional, etc) | Response: |
|  | do women receiving concerning IYCF? |  |
| 6. | In your opinion, which complementary feeding | d. Why? |
|  | recommendation is hardest for women to |  |
|  | follow? |  |

- Early Initiation of B/F (within 1 hr)
- EBF for first 6 mo
- Timely intro of CFd (after 6 mo)
- Cont'd BF for 2 yr or beyond
- Age approp CF (

Questions related to the MNP distribution and use

| 7. What do you know about the MNPs given to | | Response: |
| --- | --- | --- |
|  |
|  | mothers to add to their children’s food? |  |
| •How was the MNP Initiative introduced (How did | |  |
|  | women get to know, and start receiving the MNPs)? |  |
| •What did the mothers in the community think about | |  |
|  | the MNP program? |  |
| • | What did the health facilities staff did whilst running |  |
|  | the program? |  |
| • | What actions or approaches taken by the health |  |
|  | workers went well? |  |
| • | Which actions or approaches taken by the health |  |
|  | workers did not go well? |  |
| • | What examples of unexpected results did women |  |
|  | encounter? |  |
| • | What are women’s future expectations? |  |

2

**Lessons learned from the use of Micronutrient Powders in young child feeding in four districts in Ghana**

Appendix 4: Questionnaire for health workers

**Respondent**: Please provide a numerical rating (**1 = Not at All or Poor; 2 = Adequate or Satisfactory; and 3 = To a great extent or Excellent**) for each of the questions. As much as possible, please always provide comments on the

| Name of health facility: _______________________ | | | | | | Date (dd/mm/yyyy): ________________ | |  |
| --- | --- | --- | --- | --- | --- | --- | --- | --- |
|  | |  | |  | |  | |  |
|  | | **QUESTIONS** | | **RATING** | | **COMMENTS** | |  |
|  | | **SUMMARY** | | **OF ACTIVITIES/DECISIONS** | | | |  |
|  | |  | |  | |  | |  |
| 1) | | What sequence of activities did you perform | |  | |  | |  |
|  | | during the implementation of the project? | |  | |  | |  |
| 2) | | What key decisions did you take during the | |  | |  | |  |
|  | | implementation of the project? | |  | |  | |  |
|  | | **PROJECT EFFECTIVENESS** | | | | | |  |
| 3) | | To what extent were the objectives of the project | |  | |  | |  |
|  | | met? | |  | |  | |  |
|  | |  | |  | |  | |  |
| 4) | | What is your overall assessment of the outcome of | |  | |  | |  |
|  | | this project? | |  | |  | |  |
|  | |  | | **SCHEDULE** | | | |  |
| 5) | | How closely did the initial project schedule | |  | |  | |  |
|  | | compare with the actual schedule? | |  | |  | |  |
| 6) | | How effective were Best Practices & Lessons | |  | |  | |  |
|  | | Learned from prior projects utilized in this project? | |  | |  | |  |
|  | | **COMMUNICATION MANAGEMENT** | | | | | |  |
| 7) | | How effective were the communications materials | |  | |  | |  |
|  | | in providing and orienting district/hospital staff | |  | |  | |  |
|  | | involved in the project about the details of the | |  | |  | |  |
|  | | project? | |  | |  | |  |
| 8) | | How efficient were project team meetings | |  | |  | |  |
|  | | conducted? | |  | |  | |  |
| 9) | | How timely were progress reports provided to the | |  | |  | |  |
|  | | Project Manager by Team Members? | |  | |  | |  |
| 10) | | How actively and meaningfully were stakeholders | |  | |  | |  |
|  | | involved in the project? | |  | |  | |  |
| 11) | | Were stakeholder communications adequate and | |  | |  | |  |
|  | | effective? | |  | |  | |  |
| 12) | | How well were your expectations met regarding | |  | |  | |  |
|  | | the frequency and content of information that was | |  | |  | |  |
|  | | conveyed to you by the Project Manager? | |  | |  | |  |
| 13) | | How well were project issues communicated | |  | |  | |  |
|  | | throughout your involvement in the project? | |  | |  | |  |
| 14) | | How well did the Project Manager respond to your | |  | |  | |  |
|  | | questions or comments related to the project? | |  | |  | |  |
|  | | **ISSUE MANAGEMENT** | | | | | |  |
| 15) | | How effectively were issues managed on the | |  | |  | |  |
|  | | project? | |  | |  | |  |
| 16) | | How effectively were issues resolved before | |  | |  | |  |
|  | | escalation was necessary? | |  | |  | |  |
| 17) | | How effectively were issues able to be resolved | |  | |  | |  |
|  | | without impacting the Project Schedule? | |  | |  | |  |
|  | | **PROJECT IMPLEMENTATION & SUPPORT** | | | | | |  |
| 18) | | How effective was the documentation that you | |  | |  | |  |
|  | | received with the project implementation? | |  | |  | |  |
| 19) | | How effective was the training you received in | |  | |  | |  |
|  | | preparation for the project? | |  | |  | |  |
| 20) | | How timely was the training you received in | |  | |  | |  |
|  | | preparation for the project? | |  | |  | |  |
| 21) | | How effective was the support you received during | |  | |  | |  |
|  | | implementation of the project? | |  | |  | |  |
|  | | **PERFORMANCE OF THE PROJECT TEAM** | | | | | |  |
| 22) | | Overall, how effective was the performance of the | |  | |  | |  |
|  | | Project Manager? | |  | |  | |  |
| 23) | | How well did the Project Team understand the | |  | |  | |  |
|  | | expectations of their specific roles and | |  | |  | |  |
|  | | responsibilities? | |  | |  | |  |
| 24) | | How well were your expectations met regarding | |  | |  | |  |
|  | | the extent of your involvement in the project | |  | |  | |  |
|  | | (effort, time commitments, etc.)? | |  | |  | |  |
| 25) | How effective was each Project Team member in | |  | |  | |  | |
|  | fulfilling his/her role? | |  | |  | |  | |
|  | **GENERAL QUESTIONS** | | | | | |  | |
| 26) | What went well as planned or intended? | |  | |  | |  | |
| 27) | What did not go well as planned or intended? | |  | |  | |  | |
| 28) | What unexpected issues or events did the health | |  | |  | |  | |
|  | workers have to face? | |  | |  | |  | |
| 29) | In the event of such unexpected circumstances, | |  | |  | |  | |
|  | what useful strategies or solutions did health | |  | |  | |  | |
|  | workers develop to address the situation? | |  | |  | |  | |
| 30) | Were there any problems that could never be | |  | |  | |  | |
|  | resolved? If so, what preventative measures can | |  | |  | |  | |
|  | be developed if the project were to be started | |  | |  | |  | |
|  | again? | |  | |  | |  | |
| 31) | What would you do differently, if the project was to | |  | |  | |  | |
|  | be implemented all over again? | |  | |  | |  | |
| 32) | What were the lessons learned on this project? | |  | |  | |  | |
| 33) | Are there any “best practices” that can be | |  | |  | |  | |
|  | observed from this program? | |  | |  | |  | |
| 34) | What recommendations or advice may be offered | |  | |  | |  | |
|  | to others implementing a similar project on a pilot | |  | |  | |  | |
|  | basis and on a larger scale? | |  | |  | |  | |
| 35) | What other questions should we have asked? | |  | |  | |  | |
|  | What other information would you like to provide | |  | |  | |  | |
|  | to us about this project? | |  | |  | |  | |

**UP Template Version:** 11/30/06 Page 2 of 3

Source: CDC (https://www2.cdc.gov/cdcup/library/templates/CDC_UP_Lessons_Learned_Post_[Project_Survey.doc)](https://www2.cdc.gov/cdcup/library/templates/CDC_UP_Lessons_Learned_Post_Project_Survey.doc)
